# The association between mental health symptoms and mobility limitation among Russian, Somali and Kurdish migrants: a population based study

**DOI:** 10.1186/s12889-015-1629-1

**Published:** 2015-03-20

**Authors:** Shadia Rask, Anu E Castaneda, Päivikki Koponen, Päivi Sainio, Sari Stenholm, Jaana Suvisaari, Teppo Juntunen, Tapio Halla, Tommi Härkänen, Seppo Koskinen

**Affiliations:** National Institute for Health and Welfare, Helsinki, Finland; Department of Public Health, University of Turku, Turku, Finland; School of Health Sciences, University of Tampere, Tampere, Finland; The Psychiatric Clinic for Immigrants, Tampere, Finland

**Keywords:** Migrants, Mobility limitation, Anxiety, Depression, Somatization, Population-based study

## Abstract

**Background:**

Research has demonstrated a bidirectional relationship between physical function and depression, but studies on their association in migrant populations are scarce. We examined the association between mental health symptoms and mobility limitation in Russian, Somali and Kurdish migrants in Finland.

**Methods:**

We used data from the Finnish Migrant Health and Wellbeing Study (Maamu). The participants comprised 1357 persons of Russian, Somali or Kurdish origin aged 18–64 years. Mobility limitation included self-reported difficulties in walking 500 m or stair climbing. Depressive and anxiety symptoms were measured using the Hopkins Symptom Checklist-25 (HSCL-25) and symptoms of somatization using the somatization subscale of the Symptom Checklist-90 Revised (SCL-90-R). A comparison group of the general Finnish population was selected from the Health 2011 study.

**Results:**

Anxiety symptoms were positively associated with mobility limitation in women (Russians odds ratio [OR] 2.98; 95% confidence interval [CI] 1.28–6.94, Somalis OR 6.41; 95% CI 2.02–20.29 and Kurds OR 2.67; 95% CI 1.41–5.04), after adjustment for socio-demographic factors, obesity and chronic diseases. Also somatization increased the odds for mobility limitation in women (Russians OR 4.29; 95% CI 1.76–10.44, Somalis OR 18.83; 95% CI 6.15–57.61 and Kurds OR 3.53; 95% CI 1.91–6.52). Depressive symptoms were associated with mobility limitation in Russian and Kurdish women (Russians OR 3.03; 95% CI 1.27–7.19 and Kurds OR 2.64; 95% CI 1.39–4.99). Anxiety symptoms and somatization were associated with mobility limitation in Kurdish men when adjusted for socio-demographic factors, but not after adjusting for obesity and chronic diseases. Finnish women had similar associations as the migrant women, but Finnish men and Kurdish men showed varying associations.

**Conclusions:**

Mental health symptoms are significantly associated with mobility limitation both in the studied migrant populations and in the general Finnish population. The joint nature of mental health symptoms and mobility limitation should be recognized by health professionals, also when working with migrants. This association should be addressed when developing health services and health promotion.

**Electronic supplementary material:**

The online version of this article (doi:10.1186/s12889-015-1629-1) contains supplementary material, which is available to authorized users.

## Background

Previous studies have demonstrated a bidirectional relationship between physical function and depression. Depressed mood may either precede limitations in physical functioning or follow from deteriorating physical function, and both conditions may progress simultaneously and share aetiology [1,2]. The causal ordering between deteriorating physical function and depression is often difficult to determine, and few studies have examined the factors underlying their association. The model of depression and disability by Lenze and co-workers [3] suggests mechanisms by which depression could lead to physical disability: through increased risk for incident physical illness, poor health behaviours, and features of the depressed state (e.g. apathy and decreased pain threshold). Equally, physical disability may lead to depression through mechanisms such as social activity restriction and loss of perceived control. Other underlying factors such as medical illness may lead to both depression and physical disability. Ultimately, the mutually reinforcing relationship between depression and poor physical function may cause deteriorating health [2].

A prospective cohort study of European adults demonstrated that those with depression and/or anxiety disorder had lower levels of physical function at baseline and over time compared to those with no diagnosis, and conversely lower levels of physical function at baseline were associated with the onset of depression and/or anxiety [4]. In the US, African Americans with severe depressive symptom have been reported to have higher odds for mobility limitation than those without severe depressive symptoms [5]. Schonfeld and co-workers [6] have reported that untreated anxiety disorders and major depressive disorder are associated with significant reductions in functioning, as measured by the Short-Form Health Survey (SF-36). Using the same measure of functioning, Zayfert and co-workers [7] demonstrated that post-traumatic stress disorder (PTSD) was independently associated with impairments of physical functioning, whereas major depressive disorder was only weakly related to physical functioning.

There is inconsistent evidence on whether migrants are more vulnerable to poor mental health than non-migrants. A population-based study in 23 European countries showed that the prevalence rates of depressive symptoms are higher for migrant groups than the native population in a substantial part of European countries [8]. Other studies have demonstrated the ‘immigrant paradox’ and reported lower risk of psychiatric disorders among immigrants [9,10]. Comparing between studies is difficult due to the diversity of the studied migrants groups in terms of age, gender, country of origin and destination, socioeconomic status, and type of migration [11].

Depression and mental ill-health in migrants can be attributed to a number of possible explanations such as psychological and biological vulnerability, social skills deficit, negative life events, culture shock, and achievement expectations [12,13]. Research shows that exposure to major stressor events – such as experiences related to migration – can result in decreased psychological well-being, often in the form of poorer physical and mental health status [14-17]. In many ethno-cultural groups somatic symptoms serve as cultural idioms of distress [18].

We are unaware of previous studies on the association between mental health symptoms and physical functioning in recently migrated populations. Jørgensen and co-workers [19] searched for studies on the functioning of traumatized refugees and found none. As Fazel and co-workers [20] point out, many surveys reporting PTSD in refugees do not report the functional impairment or treatment needs associated with the disorder. The preliminary results from the Finnish Migrant Health and Wellbeing Study (Maamu) show that depressive and anxiety symptoms and limitations in physical functioning are common in Russian, Somali and Kurdish origin migrants [21], but their association has yet to be examined. Demonstrating an association between these two in three clearly defined migrant groups would add to evidence for decision-making and underline the need to address both health dimensions in the service system, also when working with migrants.

**Figure 1:**
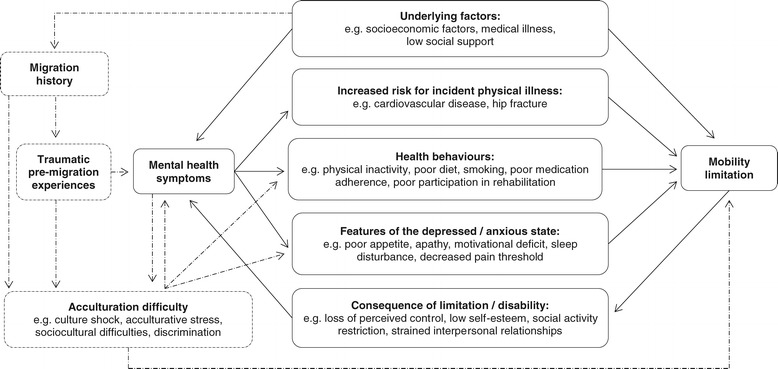
**Theoretical model of mental health symptoms and mobility limitation in the context of migrant populations.** Modification of the model of disability and depression by Lenze et al. [3], dashed lines are additions made by the authors.

Mobility limitations are often the first sign of deteriorating physical functioning, and therefore this study on working-age migrants will focus on limitations in mobility. In Figure 1 we have modified the model of Lenze and colleagues [3] to present the inter-relationship of mental health symptoms and mobility limitation in the context of migrants. The aim of the present study was 1) to determine the cross-sectional associations between mental health symptoms and mobility limitation in Russian, Somali and Kurdish origin migrants in Finland in a health survey for a random population sample, and 2) to examine whether the found associations between depressive and anxiety symptoms and mobility limitation in the three migrant groups are similar to the associations found in the general Finnish population.

## Methods

Data in the present study are from the Finnish Migrant Health and Wellbeing Study (Maamu), a comprehensive cross-sectional interview and health examination survey conducted in Finland in 2010–12 [21]. Data were collected by trained personnel of Russian, Somali, and Kurdish origin who spoke both the language of the respective target group and Finnish. The study protocol included a face-to-face interview on health and wellbeing and a health examination. A supplementary short interview or questionnaire was collected from those refusing to participate in the long interview.

### Study participants

A sample of 3000 persons was randomly selected from the National Population Registry and comprised 1000 Russian, 1000 Somali, and 1000 Kurdish origin adults aged between 18 to 64 years and living in six Finnish cities. A total of 70% of the invited Russian (n = 702), 51% of Somali (n = 512), and 63% of Kurdish (n = 632) origin persons participated in at least one part of the survey. The measures included in this study were collected in the health examination, and the participation rates to this part of the study were 47% in Russian (n = 468), 38% in Somali (n = 378), and 52% in Kurdish (n = 520) origin persons. We included those participants with data for the outcome variable and for at least one of the explanatory variables, leaving a sample of 1357 persons. A supplementary table including the participation rates by gender is provided (Additional file 1: Table S1). Descriptive statistics are presented for these 1357 persons, but Russian and Somali men had to be excluded from further analysis due to too few observations in mental health symptoms and in mobility limitation.

The inclusion criteria for Russian origin migrants was birthplace in the former Soviet Union or Russia and mother tongue Russian or Finnish, for Somali origin migrants birthplace in Somalia, and for Kurdish origin migrants birthplace in Iraq or Iran and mother tongue Kurdish. Mother tongue was used as an inclusion criterion for migrants born in the former Soviet Union and Russia to identify Russian origin migrants from other ethnic groups born in the former Soviet Union, such as Ukrainians and Uzbeks. Finnish was included as a mother tongue for the Russian sample to include Ingrians. For Iraqi and Iranian born migrants, Kurdish mother tongue was used to identify Kurdish ethnicity. Mother tongue was an inclusion criterion due to technical necessity as the interview and other study material were translated only into Russian, Somali and Kurdish languages. Interpreters were not used as data were collected by personnel who spoke both the language of the respective target group and Finnish. In addition to birthplace and mother tongue, the inclusion criteria included residence in Finland for at least a year. Persons still living in reception centres did not meet the inclusion criteria. In 2008, 93% of Somali, 67% of Kurdish, and 47% of Russian origin migrants in Finland (meeting the inclusion criteria) lived in the six municipalities included in the study. Details on the Maamu Study have been reported elsewhere [21].

Russian-speaking migrants are the largest migrant group in Finland, accounting for 23% of all foreign-speakers. Globally, Finland has the fourth largest Russian origin population outside the former Soviet Union [22]. Migrants from Russia have generally moved to Finland voluntarily, as much of this migration is explained by the return migration of the Ingrians, personal relationships (e.g. marriage to Finnish spouse) and labour migration. Somali origin migrants are the fourth largest migrant group, and the largest refugee and pre-dominantly muslim-faithed migrant group in Finland. Kurdish-speaking migrants are the sixth largest migrant group, and Iraqi and Iranian refugees have been among the largest quota refugee groups accepted to Finland in recent years. [23] Contrary to Russian-speaking migrants, most Kurdish and Somali origin migrants have moved to Finland as refugees, asylum seekers or based on family reunion. These three migrant groups were selected to represent potentially vulnerable groups from different geographical areas and the two latter with a high proportion of migrants with refugee status.

Russian, Somali and Kurdish origin migrants are significant also from an international perspective. Somalis have been one of the main groups of migrants moving to Scandinavia (Sweden, Norway and Denmark) in the 2000s [24]. Also Iraqi and Iranian migrants, including Kurds, have been dominant groups migrating to Sweden and Denmark [24]. Migrants from Somalia, Iraq and Iran are significant ethnic groups also outside Scandinavia in countries such as the United States, Canada, Australia, the United Kingdom, and Germany. There are also significant Russian-speaking communities in Germany, the United States, and Canada, but also in countries such as Israel. The Russian-speaking population living outside of Russia amounts to 25–30 million [22]. Also in other Nordic countries, Sweden and Norway, Russian origin migrants are among the 15 largest migrant groups [24].

A comparison group of the general Finnish population was selected from the national sample of the Health 2011 study [25], including all sampled persons within the age range 30–64 years who participated in the survey and living in the same municipalities as selected for the Maamu Study (n = 892).

### Ethical approval

The Maamu Study was approved by the Coordinating Ethical Committee of the Helsinki and Uusimaa Hospital Region, Finland. Written informed consent was obtained from each participant.

### Measures

#### Mobility limitation

As part of the health examination and the short interview, participants were asked: “Can you walk 0.5 km without resting?” and “Can you climb up several flights of stairs without resting?” [26]. The response categories were: without difficulties, with minor difficulties, with major difficulties, or not at all. We used the same classification of mobility limitation as Stenholm and co-workers [27]: participants reporting any difficulties in walking 500 m or stair climbing were considered to have mobility limitation. The series of interview questions are originally based on the ADL and IADL measures developed by Katz et al. [28,29], Lawton and Brody [30] and OECD [26], which were later modified and complemented for the Health 2000 Survey [31] on the basis of experiences from the Mini-Finland Health Examination Survey [32]. The Health 2011 study included the measure of mobility for participants aged 30 and above.

#### Mental health symptoms

As part of the health examination, the Hopkins Symptom Checklist-25 (HSCL-25) [33] was used to measure symptoms of depression and anxiety in a self-administered questionnaire (some participants were interviewed due to difficulties in reading). HSCL-25 is a shortened version of a 90-item questionnaire designed by Derogatis et al. [34], and it is a cross-culturally valid instrument [35,36]. This version includes 15 items on the occurrence of depressive symptoms and 10 items on anxiety symptoms during the past seven days (Table 1). The scale ranges from 1 (“not at all”) to 4 (“extremely”). We calculated mean scores for the depression (HSCL-15) and anxiety (HSCL-10) subscales separately. Responses were summed and divided by the number of answered items to generate a symptoms mean score ranging from 1.0 to 4.0. Participants were included in the analysis if they had responded to at least 11 items of the HSCL-15, and 8 items of the HSCL-10. There are two commonly used cut-off points for prevalent depressive and anxiety symptoms: 1.55 and 1.75 [37]. The results were similar by using these two cut-off values, and therefore only results for cut-off score 1.75 are presented.

**Table 1:** **Items in the depression, anxiety and somatization subscales**

**Item** ^*^	**Subscale**
Loss of sexual interest or pleasure (5)	DEP
Feeling low in energy, slowed down (14)	DEP
Thoughts of ending one’s life (15)	DEP
Poor appetite (19)	DEP^1^
Crying easily (20)	DEP
Feeling trapped or caught (22)	DEP
Blaming oneself for things (26)	DEP
Feeling lonely (29)	DEP
Feeling blue (30)	DEP
Worrying too much about things (31)	DEP
Feeling no interest in things (32)	DEP
Difficulty falling asleep or staying asleep (66)	DEP^1^
Feeling hopeless about the future (54)	DEP
Feeling everything is an effort (71)	DEP
Feelings of worthlessness (79)	DEP
Nervousness or shakiness inside (2)	ANX
Trembling (17)	ANX
Being suddenly scared for no apparent reason (23)	ANX
Feeling fearful (33)	ANX
Heart pounding or racing (39)	ANX
Feeling tense or keyed up (57)	ANX
Spells of terror or panic (72)	ANX
Feeling restless, not being able to sit still (78)	ANX
Headaches (1)	ANX^2^, SOM
Faintness, dizziness, or weakness (2)	ANX^2^, SOM
Pains in heart or chest (12)	SOM
Pains in lower back (27)	SOM
Nausea or upset stomach (40)	SOM
Soreness of muscles (42)	SOM
Trouble getting your breath (48)	SOM
Hot or cold spells (49)	SOM
Numbness or tingling in parts of your body (52)	SOM
A lump in your throat (53)	SOM
Feeling weak in parts of your body (56)	SOM
Heavy feelings in your arms or legs (58)	SOM

DEP = Hopkins Symptom Checklist-25 [21], depression subscale, ANX = Hopkins Symptom Checklist-25 [21], anxiety subscale, SOM = Symptom Checklist-90 [22], somatization subscale.

^*^In brackets the original numbers from the SCL-90 [22].

^1^Included in the depression scale of the HSCL-25, but not the depression scale of the SCL-90.

^2^Included in the anxiety scale of the HSCL-25, but not the anxiety scale of the SCL-90.

Symptoms of somatization were assessed using the somatization subscale of the SCL-90-R [34]. The subscale includes 12 items on the occurrence of somatic symptoms during the past seven days (Table 1), and the response categories are identical with those of the HSCL-25. Participants who responded being at least a little bothered by eight or more symptoms were considered to have symptoms of somatization [38]. To be included in analysis, participants had to have responded to at least eight items. Analysis including the Finnish general population could only be conducted for depressive and anxiety symptoms as the Health 2011 data did not include information on somatization.

### Control variables

Factors which were known or expected to be related to mental health symptoms and mobility limitation were examined separately for the three ethnic groups. Economic situation, unemployment, lifestyle factors, social relations and country of origin have been demonstrated to be associated with mental health symptoms [39]. Obesity, smoking, and somatic diseases are known to be associated with mobility limitation in the Finnish general population [40]. Based on literature [41-43] and the theoretical model of mental health symptoms and mobility limitation (Figure 1), we investigated the relationships of the following variables with mental health symptoms and mobility limitation: age, basic education, vocational training, employment, economic situation, time lived in Finland, age of moving to Finland, language proficiency in Finnish/Swedish (the two main official languages of Finland), refugee status, chronic diseases, obesity, injuries, smoking, regular alcohol consumption, and loneliness. Only those variables that were statistically significantly associated (chi-square test P <0.05) with both mental health symptoms and mobility limitation were used in the main analyses. To keep the number of adjusted variables reasonable, if two or more variables characterizing the same phenomenon (e.g. basic education and vocational training) were each statistically significantly associated with mental health symptoms and mobility limitation, we chose the variable with the strongest associations or in some cases (e.g. time lived in Finland, age of moving to Finland and language proficiency) the most meaningful proxy.

The final socio-demographic variables adjusted in the main analyses were age, education, economic situation and language proficiency in Finnish/Swedish. We used a continuous variable for age. Education level was dichotomised into high school graduates (or having completed part of high school) and having less than high school education. Economic situation was assessed by asking “considering the total income of your household, how difficult or easy is it to cover your costs”. The response categories were dichotomised into fairly easy, easy or very easy and very difficult, difficult or fairly difficult. Language proficiency in Finnish/Swedish was included in the main analyses as a proxy for adjustment to the Finnish society. Language proficiency was assessed by asking how well the participant understands Finnish/Swedish. The response categories were dichotomised into not at all or poorly and moderately or well.

The final health-related variables that we adjusted for were obesity (body mass index (BMI), ≥30 kg/m2) and selected chronic conditions. BMI was based on measured height and weight. We formed one dichotomised variable for selected chronic conditions which included chronic injury, cardiovascular disease, asthma, chronic bronchitis, knee osteoarthritis or hip osteoarthritis, as some of these conditions were too rare to be analysed separately. Those reporting at least one of the selected conditions were categorized as having a chronic condition. Injuries were self-reported, and other physical conditions were self-reports of conditions ever diagnosed by a physician.

### Statistical analysis

Age-adjusted prevalence and means were calculated by gender in the ethnic groups using predicted margins [44]. Inverse probability weights (IPW) [45] calculated with age group, gender, ethnic group, municipality and marital status were used to account for the different sampling probabilities, to reduce the effects of non-response, and to produce estimates for means and percentages that are representative of Russian, Somali, and Kurdish migrants in Finland. The population sizes were relatively small, and a significant proportion of the total population was included in the sample, and thus the finite population correction [46] was applied in all analyses.

The interaction between gender and mental health on mobility limitation was tested, and because significant interactions were found, the analyses were stratified by gender. The associations between mental health symptoms and mobility limitation were calculated using logistic regression analysis. Four models were constructed, including an increasing number of adjusted variables. In Model 1, the relation between each mental health symptom set (symptoms of depression, anxiety and somatization) and mobility limitation was examined adjusting only for age. In Model 2, age, education and economic situation were adjusted for. Model 3 was exactly the same as Model 2, but included also language proficiency. Model 4 included also BMI and chronic conditions. In the analysis including the Finnish comparison group, age and education were adjusted for. The results are presented as odds ratios (OR) with 95% confidence intervals (CI). All analyses were conducted using SAS 9.3/SUDAAN 11.0.0 software, which takes into account the sampling design [47]. P < 0.05 was considered as statistically significant.

## Results

The main characteristics of the study population are presented in Table 2.

**Table 2:** **Descriptive statistics of the study population by gender**

	**Russian (n = 467)**		**Somali (n = 378)**		**Kurdish (n = 512)**	
**Characteristics**	**Men (n = 167)**	**Women (n = 300)**	**Men (n = 155)**	**Women (n = 223)**	**Men (n = 275)**	**Women (n = 237)**
	**%** ^**1**^ **(n)** ^**2**^	**%** ^**1**^ **(n)** ^**2**^	**%** ^**1**^ **(n)** ^**2**^	**%** ^**1**^ **(n)** ^**2**^	**%** ^**1**^ **(n)** ^**2**^	**%** ^**1**^ **(n)** ^**2**^
Age (mean, SE)	36.6, 1.1 (167)	40.8, 0.8 (300)	32.8, 1.0 (155)	35.3, 0.8 (223)	35.0, 0.6 (275)	35.5, 0.6 (237)
High school graduate^3^	73.9 (117)	83.9 (250)	42.1 (47)	16.2 (29)	40.5 (103)	42.6 (96)
Good economic situation	60.0 (93)	47.0 (146)	40.6 (44)	47.5 (74)	35.8 (91)	30.9 (71)
Unemployed	26.6 (44)	26.2 (70)	29.8 (40)	21.3 (43)	27.6 (75)	25.3 (62)
Chronic conditions^4^	22.5 (46)	30.0 (90)	5.7 (9)	26.9 (49)	29.8 (80)	35.5 (85)
Obese (BMI ≥ 30)^5^	12.3 (21)	17.4 (53)	2.7 (8)	36.2 (77)	14.6 (41)	22.3 (52)
Poor language proficiency in Finnish/Swedish	16.1 (30)	9.3 (31)	7.7 (18)	28.2 (65)	9.5 (30)	18.1 (43)
Time in Finland (mean, SE)	11.3, 0.5 (163)	12.3, 0.4 (298)	11.9, 0.6 (144)	11.5, 0.4 (208)	10.4, 0.3 (263)	11.3, 0.3 (236)
Refugee or asylum seeker	1.2 (2)	1.2 (2)	92.9 (111)	52.4 (95)	84.8 (212)	63.4 (141)
Depressive symptoms^a^	12.0 (17)	23.5 (65)	6.3 (10)	12.5 (26)	26.3 (65)	49.2 (117)
Anxiety symptoms^b^	4.0 (7)	23.2 (67)	4.1 (9)	8.3 (22)	20.0 (49)	42.5 (101)
Somatization^c^	5.2 (9)	22.0 (63)	7.8 (15)	15.8 (38)	20.9 (53)	38.8 (94)
Mobility limitation^d^	4.6 (10)	15.7 (47)	8.3 (18)	33.3 (80)	19.4 (57)	42.3 (103)

^1^weighted prevalence, weighted mean for age and time lived in Finland.

^2^crude n.

^3^Has completed high school or part of high school in any country.

^4^Those reporting injuries and/or at least one of the following self-reported diseases ever diagnosed by a physician: cardiovascular disease (coronary artery disease, high blood pressure, and diabetes), asthma, chronic bronchitis, knee osteoarthritis or hip osteoarthritis.

^5^BMI = Body mass index (kg/m^2^).

^a^HSCL-25 (Hopkins Symptom Checklist-25) [21], depression subscale, cut-off point > 1.75.

^b^HSCL-25 (Hopkins Symptom Checklist-25) [21], anxiety subscale, cut-off point > 1.75.

^c^SCL-90 (Symptom Checklist-90) [22], somatization subscale, cut-off point 8/12 symptoms.

^d^Self-reported difficulties in walking 500 m or stair climbing.

### Depressive symptoms

Depressive symptoms increased the odds for mobility limitation in women in all migrant groups, but not in Kurdish men, in the age-adjusted model (Table 3). Controlling for education and economic situation in Model 2 attenuated the strength of the associations in Russian and Somali women to non-significance. With the addition of language proficiency in Model 3, the association in Russian women was strengthened to statistical significance. In the fully adjusted model controlling for socio-demographic factors and health-related variables, depressive symptoms remained significantly associated with mobility limitation in Russian and Kurdish women. The associations between each of the adjusted variables and mobility limitation are presented in Table 4.

**Table 3:** **Mental health-related factors associated with mobility limitation**

	**Men** ^**†**^	**Women**		
	**Kurdish**	**Russian**	**Somali**	**Kurdish**
**Explanatory variable**	**OR** ***(95% CI)***	**OR** ***(95% CI)***	**OR** ***(95% CI)***	**OR** ***(95% CI)***
**Depressive symptoms** ^**a**^				
Model 1	1.82 (0.93–3.57)	**2.89 (1.32–6.31)**	**4.67 (1.95–11.20)**	**2.56 (1.52–4.31)**
Model 2	1.74 (0.87–3.46)	2.18 (0.98–4.88)	2.57 (1.00–6.61)	**2.56 (1.47–4.44)**
Model 3	1.71 (0.85–3.44)	**2.51 (1.11–5.68)**	2.53 (0.97–6.56)	**2.50 (1.44–4.34)**
Model 4	1.66 (0.82–3.33)	**3.03 (1.27–7.19)**	2.53 (0.98–6.53)	**2.64 (1.39–4.99)**
**Anxiety symptoms** ^**b**^				
Model 1	**2.33 (1.11–4.85)**	**3.00 (1.39–6.46)**	**8.78 (3.00–25.74)**	**3.09 (1.83–5.23)**
Model 2	**2.26 (1.07–4.80)**	**2.56 (1.18–5.57)**	**6.07 (1.84–20.04)**	**2.92 (1.68–5.06)**
Model 3	**2.23 (1.05–4.75)**	**2.85 (1.31–6.18)**	**6.16 (1.94–19.55)**	**2.80 (1.60–4.90)**
Model 4	2.16 (0.98–4.76)	**2.98 (1.28–6.94)**	**6.41 (2.03–20.29)**	**2.67 (1.41–5.04)**
**Somatization** ^**c**^				
Model 1	**2.27 (1.24–4.14)**	**4.82 (2.15–10.80)**	**11.28 (4.59–27.75)**	**3.80 (2.23–6.46)**
Model 2	**2.14 (1.16–3.98)**	**4.09 (1.80–9.30)**	**16.10 (5.31–48.83)**	**3.99 (2.30–6.90)**
Model 3	**2.12 (1.14–3.97)**	**4.57 (2.00–10.41)**	**16.38 (5.52–48.60)**	**4.00 (2.30–6.96)**
Model 4	1.88 (0.96–3.68)	**4.29 (1.76–10.44)**	**18.83 (6.15–57.61)**	**3.53 (1.91–6.52)**

^†^Russian and Somali men were excluded from the analysis due to too few observations.

OR = odds ratio, bolded ORs represent significant associations.

95% CI = 95% confidence interval.

^a^HSCL-25 (Hopkins Symptom Checklist-25) [21], depression subscale, cut-off point > 1.75.

^b^HSCL-25 (Hopkins Symptom Checklist-25) [21], anxiety subscale, cut-off point > 1.75.

^c^SCL-90 (Symptom Checklist-90) [22], somatization subscale, cut-off point 8/12 symptoms

Model 1 = adjusted for age.

Model 2 = adjusted for age, education, and economic situation.

Model 3 = adjusted for age, education, economic situation, and Finnish/Swedish language proficiency.

Model 4 = adjusted for age, education, economic situation, Finnish/Swedish language proficiency, BMI, and selected chronic conditions (those reporting injuries and/or at least one of the following self-reported diseases ever diagnosed by a physician: cardiovascular disease (coronary artery disease, high blood pressure, and diabetes), asthma, chronic bronchitis, knee osteoarthritis or hip osteoarthritis).

**Table 4:** **Mental health-related factors and background factors** ^**1**^ **associated with mobility limitation**

	**Men** ^**†**^	**Women**		
	**Kurdish**	**Russian**	**Somali**	**Kurdish**
**Explanatory variable**	**OR** ***(95% CI)***	**OR** ***(95% CI)***	**OR** ***(95% CI)***	**OR** ***(95% CI)***
**Depressive symptoms** ^**a**^				
No	1.00	1.00	1.00	1.00
Yes	1.66 (0.82–3.33)	**3.03 (1.27–7.19)**	2.53 (0.98–6.53)	**2.64 (1.39–4.99)**
Age	**1.10 (1.06–1.14)**	**1.07 (1.03–1.12)**	**1.05 (1.01–1.10)**	**1.04 (1.01–1.08)**
Education				
Less than high school	**2.42 (1.20–4.89)**	0.78 (0.29–2.05)	**7.75 (1.67–35.91)**	1.52 (0.83–2.79)
High school graduate	1.00	1.00	1.00	1.00
Economic situation				
Fairly difficult or poorer	1.00 (0.47–2.13)	2.10 (0.90–4.91)	1.54 (0.67–3.54)	0.65 (0.33–1.28)
Fairly easy or better	1.00	1.00	1.00	1.00
Language proficiency				
Not at all or poorly	1.01 (0.38–2.69)	3.24 (0.49–21.48)	0.75 (0.28–1.96)	1.00 (0.44–2.26)
Moderately or well	1.00	1.00	1.00	1.00
BMI				
<30 kg/m^2^	1.00	1.00	1.00	1.00
≥30 kg/m^2^	1.82 (0.83–3.98)	**4.15 (1.78–9.67)**	1.28 (0.55–2.98)	**2.36 (1.13–4.91)**
Chronic conditions^2^				
None of the selected conditions	1.00	1.00	1.00	1.00
At least one selected condition	**2.04 (1.03–4.07)**	1.11 (0.44–2.80)	1.14 (0.47–2.81)	**3.13 (1.56–6.30)**
**Anxiety symptoms** ^**b**^				
No	1.00	1.00	1.00	1.00
Yes	2.16 (0.98–4.76)	**2.98 (1.28–6.94)**	**6.41 (2.03–20.29)**	**2.67 (1.41–5.04)**
Age	**1.11 (1.07–1.15)**	**1.07 (1.03–1.12)**	**1.06 (1.01–1.10)**	**1.05 (1.02–1.09)**
Education				
Less than high school	**2.50 (1.23–5.10)**	0.79 (0.32–1.96)	**8.18 (1.70–39.44)**	1.59 (0.87–2.91)
High school graduate	1.00	1.00	1.00	1.00
Economic situation				
Fairly difficult or poorer	1.01 (0.48–2.13)	2.47 (1.00–6.08)	1.27 (0.53–3.01)	0.75 (0.38–1.46)
Fairly easy or better	1.00	1.00	1.00	1.00
Language proficiency				
Not at all or poorly	0.99 (0.37–2.69)	2.86 (0.59–13.89)	0.70 (0.27–1.82)	1.00 (0.44–2.30)
Moderately or well	1.00	1.00	1.00	1.00
BMI				
<30 kg/m^2^	1.00	1.00	1.00	1.00
≥30 kg/m^2^	1.89 (0.87–4.12)	**4.07 (1.72–9.62)**	1.33 (0.57–3.13)	**2.23 (1.10–4.55)**
Chronic conditions^2^				
None of the selected conditions	1.00	1.00	1.00	1.00
At least one selected condition	1.93 (0.94–3.99)	1.14 (0.45–2.88)	1.18 (0.48–2.91)	**2.63 (1.31–5.28)**
**Somatization** ^**c**^				
No	1.00	1.00	1.00	1.00
Yes	1.88 (0.96–3.68)	**4.29 (1.76–10.44)**	**18.83 (6.15–57.61)**	**3.53 (1.91–6.52)**
Age	**1.10 (1.06–1.14)**	**1.07 (1.02–1.12)**	**1.06 (1.01–1.10)**	**1.04 (1.01–1.08)**
Education				
Less than high school	**2.19 (1.10–4.36)**	0.86 (0.37–2.04)	**13.05 (2.45–69.48)**	1.67 (0.92–3.01)
High school graduate	1.00	1.00	1.00	1.00
Economic situation				
Fairly difficult or poorer	1.11 (0.54–2.27)	2.35 (0.94–5.89)	1.01 (0.41–2.50)	0.78 (0.31–1.71)
Fairly easy or better	1.00	1.00	1.00	1.00
Language proficiency				
Not at all or poorly	1.09 (0.42–2.83)	2.79 (0.65–11.87)	0.67 (0.24–1.92)	0.73 (0.31–1.71)
Moderately or well	1.00	1.00	1.00	1.00
BMI				
<30 kg/m^2^	1.00	1.00	1.00	1.00
≥30 kg/m^2^	1.66 (0.76–3.65)	**3.66 (1.54–8.69)**	1.61 (0.63–4.08)	**2.20 (1.06–4.58)**
Chronic conditions^2^				
None of the selected conditions	1.00	1.00	1.00	1.00
At least one selected condition	1.84 (0.89–3.81)	1.06 (0.40–2.79)	0.90 (0.32–2.48)	**2.74 (1.40–5.40)**

^1^Background factors include age, education, economic situation, Finnish/Swedish language proficiency, BMI, and certain chronic conditions.

^2^Those reporting injuries and/or at least one of the following self-reported diseases ever diagnosed by a physician: cardiovascular disease (coronary artery disease, high blood pressure, and diabetes), asthma, chronic bronchitis, knee osteoarthritis or hip osteoarthritis.

^†^Russian and Somali men were excluded from the analysis due to too few observations.

OR = odds ratio, bolded ORs represent significant associations.

95% CI = 95% confidence interval.

^a^HSCL-25 (Hopkins Symptom Checklist-25) [21], depression subscale, cut-off point > 1.75.

^b^HSCL-25 (Hopkins Symptom Checklist-25) [21], anxiety subscale, cut-off point > 1.75.

^c^SCL-90 (Symptom Checklist-90) [22], somatization subscale, cut-off point 8/12 symptoms.

BMI = Body mass index (kg/m^2^).

### Anxiety symptoms

Anxiety symptoms increased the odds for mobility limitation in all migrant groups in the age-adjusted model (Table 3). The addition of education and economic situation in Model 2 decreased all associations slightly. The addition of language proficiency in Model 3 made little change to the associations. In the fully adjusted model, anxiety symptoms remained associated with mobility limitation in all groups of women, but not in Kurdish men. The associations between each of the adjusted variables and mobility limitation are presented in Table 4.

### Somatization

Somatization increased the odds for mobility limitation in all migrant groups in the age-adjusted model (Table 3). Controlling for socio-demographic variables in Model 2 had little effect on the associations in Kurds and Russian women, but the strength of the association increased somewhat for Somali women. The addition of language proficiency in Model 3 made little difference to the associations. In the fully adjusted model, somatization remained associated with mobility limitation in all groups of women, but not in Kurdish men. The associations between each of the adjusted variables and mobility limitation are presented in Table 4.

### Similarity to the general Finnish population

The main characteristics of the study population aged 30–64 years are presented in Table 5. When adjusting for age and education, depressive symptoms increased the odds for mobility limitation in all women (Table 6). Anxiety symptoms increased the odds for mobility limitation in Russian and Kurdish women, but not for Somalis. An association between anxiety symptoms and mobility limitation was found also in women in the general Finnish population. There was more dissimilarity between Kurdish men and men in the general Finnish population: depressive symptoms were associated with mobility limitation among men in the general Finnish population, but not Kurdish men, while the opposite was true for anxiety symptoms.

**Table 5:** **Descriptive statistics of the study population aged 30–64 years by gender**

	**Russian (n = 344)**		**Somali (n = 229)**		**Kurdish (n = 348)**		**Finnish (n = 892)**	
**Characteristics**	**Men (n = 119)**	**Women (n = 225)**	**Men (n = 85)**	**Women (n = 144)**	**Men (n = 181)**	**Women (n = 167)**	**Men (n = 396)**	**Women (n = 496)**
	**%** ^**1**^ **(n)** ^**2**^	**%** ^**1**^ **(n)** ^**2**^	**%** ^**1**^ **(n)** ^**2**^	**%** ^**1**^ **(n)** ^**2**^	**%** ^**1**^ **(n)** ^**2**^	**%** ^**1**^ **(n)** ^**2**^	**%** ^**1**^ **(n)** ^**2**^	**%** ^**1**^ **(n)** ^**2**^
Age (mean, SE)	44.3, 1.1 (119)	46.7, 0.7 (225)	40.6, 1.1 (85)	42.1, 0.7 (144)	41.4, 0.5 (181)	40.8, 0.5 (167)	47.2, 0.7 (396)	46.3, 0.5 (496)
High school graduate^3^	81.2 (90)	84.6 (184)	53.7 (37)	12.3 (17)	44.2 (77)	37.3 (66)	56.3 (222)	68.6 (334)
Depressive symptoms^a^	12.3 (12)	25.7 (55)	1.5 (2)	12.2 (14)	24.2 (42)	58.7 (93)	12.5 (38)	7.2 (37)
Anxiety symptoms^b^	3.9 (5)	25.5 (54)	4.2 (5)	7.1 (10)	17.7 (30)	49.4 (78)	6.3 (21)	4.1 (20)
Mobility limitation^c^	7.0 (9)	17.8 (44)	15.4 (13)	47.1 (63)	32.8 (49)	59.1 (88)	5.9 (30)	12.6 (69)

^1^weighted prevalence, weighted mean for age.

^2^crude n.

^3^Has completed high school or part of high school in any country.

^a^HSCL-25 (Hopkins Symptom Checklist-25) [21], depression subscale, cut-off point > 1.75.

^b^HSCL-25 (Hopkins Symptom Checklist-25) [21], anxiety subscale, cut-off point > 1.75.

^c^Self-reported difficulties in walking 500 m or stair climbing.

**Table 6:** **The association between depressive or anxiety symptoms and mobility limitation among Russian, Somali and Kurdish migrants and the general Finnish population (aged 30–64 yrs)** ^**1**^

	**Men** ^**†**^		**Women**			
	**Kurdish**	**Finnish**	**Russian**	**Somali**	**Kurdish**	**Finnish**
**Explanatory variable**	**OR** **(** ***95% CI*** **)**	**OR** **(** ***95% CI*** **)**	**OR** **(** ***95% CI*** **)**	**OR** **(** ***95% CI*** **)**	**OR** **(** ***95% CI*** **)**	**OR** **(** ***95% CI*** **)**
Depressive symptoms^a^	1.85 (0.87–3.94)	**4.58 (1.55–13.53)**	**2.78 (1.18–6.54)**	**4.90 (1.08–22.22)**	**2.74 (1.49–5.05)**	**4.34 (1.87–10.08)**
Anxiety symptoms^b^	**2.74 (1.15–6.54)**	3.32 (0.84–13.12)	**2.64 (1.16–6.01)**	6.98 (0.99–49.09)	**3.20 (1.74–5.90)**	**8.08 (3.05–21.43)**

^1^Adjusted for age and education.

^†^Russian and Somali men were excluded from the analysis due to too few observations.

OR = odds ratio, bolded ORs represent significant associations.

95% CI = 95% confidence interval.

^a^HSCL-25 (Hopkins Symptom Checklist-25) [21], depression subscale, cut-off point > 1.75.

^b^HSCL-25 (Hopkins Symptom Checklist-25) [21], anxiety subscale, cut-off point > 1.75.

## Discussion

The results from the Finnish Migrant Health and Wellbeing Study show that mental health symptoms are significantly associated with mobility limitation in Russian, Somali and Kurdish women, and that these associations are similar in migrant women and women in the general Finnish population. In men, the prevalence of mental health symptoms and mobility limitation was too low to study their association in Russian and Somali men. In Kurdish men and men in the general Finnish population, some associations between mental health symptoms and mobility limitation were found.

Our results are consistent with previous research showing the association between mental health symptoms and mobility limitation [1,2,4,5]. These earlier studies have, however, been conducted in the general population or among African Americans. Therefore, the present study provides new information on the association between mental health symptoms and mobility limitation in migrants. We found that mental health symptoms increased the odds for mobility limitation particularly in women. This is in agreement with previous studies: women are known to have higher rates of depression than men [48] and particularly refugee women from low-income countries have been shown to be at risk of mental ill-health [41].

Thorpe and colleagues found that depressive symptoms increased the odds for mobility limitation in African Americans for both men and women [5]. Contrary to this, we did not find consistent associations between mental health symptoms and mobility limitation in Kurdish men and we were unable to study these associations in Russian and Somali men. A potential reason for the missing association in Kurdish men is that the used indicator of mobility limitation was too easy for working-age men. Our results highlight the distress of Kurdish women, among whom the association between mental health symptoms and mobility limitation was very consistent. This is supported by previous studies reporting a high prevalence of depressive and anxiety symptoms in Iraqi and Iranian migrants [49-51], and findings that physical inactivity is strongly associated with anxiety and depression in Iraqi migrants in Sweden [52].

The differences in the associations between mental health symptoms and mobility limitation demonstrate the heterogeneity of the studied migrant groups. On the other hand, particularly in women, the associations between mental health symptoms and mobility limitation point to a similar direction in the migrant groups and women in the general Finnish population. Thus, our study suggests that mental health symptoms and mobility limitation are intertwined both in the studied migrant populations and in the general Finnish population. There are, however, several reasons why the association between mental health symptoms and mobility limitation is particularly important to consider when discussing the health of migrants. Firstly, the preliminary findings from the Finnish Migrant Health and Wellbeing Study (Maamu) show that mental health symptoms are significantly more common among Russian women and Kurdish men and women compared to the general Finnish population [21]. Also limitations in physical functioning are more common among Somali women and Kurdish men and women compared to the general Finnish population [21]. Another important aspect, which differentiates migrant populations from the general population, is that migrants are known to have more barriers in access to health care than the general population [53]. Migrants facing mental health symptoms and limitations in mobility may be less likely – due to a range of reasons such as language barriers, lack of knowledge, fear, poverty and stigma – to seek and receive adequate care than the general population [54,55].

We found that socio-demographic and health-related factors could not fully explain the associations between mental health symptoms and mobility limitation in migrant women. This is in agreement with a previous study demonstrating that the risk of depression associated with migrant status cannot be fully explained by socio-demographic factors [43]. However, causal relations between mental health symptoms and mobility limitation cannot be confirmed in our cross-sectional study. Previous studies have demonstrated that various socio-economic factors from childhood and midlife independently predict both mobility limitation and depressed mood in old age [56], and that those with low education have an increased risk of self-estimated running difficulties [57] and mobility limitation [40] than those with high education. Also life satisfaction and positive judgments about the future are known to contribute to better functional status [58].

Adding to the model of Lenze and colleagues [3], we hypothesize that acculturation difficulty may be a pathway from migration history to mobility limitation (Figure 1). Acculturative stress and difficulties, for instance, in finding housing or employment may lead to apathy and functional decline. Also the unfamiliarity of the new environment and discrimination may hinder activities and promote sedentary lifestyle, leading to mobility limitation through poor health behaviours. Studies show that somatization may be a coping mechanism to the stress of acculturation and may be caused by a difficult period of adjustment [59,60]. Supporting our hypothesis, we found an association between anxiety symptoms and mobility limitation in Kurdish men, but not men in the general Finnish population. However, in women, the association between anxiety symptoms and mobility limitation was present both in the general Finnish population and the migrant populations, which suggests that there may be variations in the factors underlying the found associations. The strong association between anxiety symptoms and mobility limitation suggests that features of the anxious state such as feeling fearful may contribute to perceived difficulties in mobility.

Our study provides limited information on the role of health behaviour in the association between mental health symptoms and mobility limitation as smoking was not associated with mental health symptoms or mobility limitation. We also excluded physical activity from our analysis because the cross-sectional study design would have made it difficult to interpret associations between physical activity and mobility limitation, as physical inactivity can both precede and follow from mobility limitation. Previous studies have, however, shown that persons with depressed mood are less active, and their sedentary lifestyle may cause functional decline [1,61]. A high prevalence of physical inactivity and obesity has been reported in Iranian migrants in Sweden [62-65] and Somali women in Norway [66]. Cultural influences in body size preference and less value placed on physical activity may emphasize sedentary lifestyle in migrant women [42].

Important strengths of our study are the population-based study design and the relatively high participation rate compared to other migrant health studies. There are also some limitations. Although the participation rate was satisfactory, it is generally known that the effects of non-response cannot be completely corrected for, particularly for Somalis among whom the participation rate was lowest. We have aimed to correct this using of Inverse Probability Weighting (IPW, [45,67]), which is commonly utilized in correcting the effects of non-response. Another limitation is that due to lacking comparable data, we were unable to make comparisons between the migrant populations and the general Finnish population for the whole age group in concern and for all the studied mental health symptoms.

Other limitations are the validity and reliability of the used instruments in the study populations. Cross-cultural assessments of the validity of the HSCL have been conducted [35,68]. Kuittinen and colleagues have also examined the manifestation of somatic-affective and cognitive depressive symptoms among older Somali refugees and native Finns and included the SCL-90 somatization subscale in their study [69]. Even so, the low prevalence of mental health symptoms in Somalis raises questions, as mental disorders are known to have severe social stigma in the Somali community [70]. We have not found reports of cross-cultural validation of the interview questions on mobility, and we were unable to include such validation in this study. Self-reported activity limitations may be inaccurate if the respondent does not routinely undertake the asked activities [71]. It may be that Somali and Kurdish women do not regularly climb several flights of stairs, and thus overestimate difficulties in this task. Due to response tendency, participants with mental health symptoms may have assessed their mobility more pessimistically than participants without mental health symptoms. In contrast, the asked activities were fairly easy for working-age population, which may be a reason for the low prevalence of mobility limitation in men. Also, reporting difficulties in mobility may be considered socially undesirable particularly by men. Due to the low prevalence of mobility limitation in men, we had to exclude Russian and Somali men from further analysis, which limits the generalizability of the results. What can, however, be inferred from our study is that self-reported mental health symptoms and mobility limitation are uncommon among working-age Russian and Somali men. The low prevalence of mobility limitation and the sample size of the study also lead to wide confidence intervals. Lastly, the cross-sectional study design does not permit inferring causal relationships, and therefore longitudinal studies are needed. Future prospective research is needed to confirm the association and clarify the pathways through which mental health is related to physical functioning in migrant populations.

## Conclusions

This study has shown that mental health symptoms and mobility limitation are intertwined both in the studied migrant populations and in the general Finnish population. The found associations could not be fully explained by socio-demographic and health-related factors. The joint nature of mental health symptoms and mobility limitation should be recognized by health professionals, also when working with migrants. Migrants are known to have more barriers in access to health care than the general population, and therefore the association between mental health symptoms and mobility limitation needs to be addressed when developing health services and health promotion.

## Additional file

Additional file 1: Table S1. The Study Population and Participation Rates by Gender.
