# Supplementary material for: The association between mental health symptoms and mobility limitation among Russian, Somali and Kurdish migrants: a population based study
Source: BMC Public Health. 2015 Mar 20;15:275. doi: 10.1186/s12889-015-1629-1 (PMC4377023; doi:10.1186/s12889-015-1629-1)
Supplement: Additional file 1: Table S1. — The Study Population and Participation Rates by Gender. [file 12889_2015_1629_MOESM1_ESM.pdf]

Supplementary Table 1 The Study Population and Participation Rates by Gender

	Russian<br>% (N)	Somali<br>% (N)	Kurdish<br>% (N)	Total<br>(N)
At least one part of the survey[1]	70.2 (702)<br>M: 36.6 (257)<br>F: 63.4 (445)	51.2 (512)<br>M: 44.5 (228)<br>F: 55.5 (284)	63.2 (632)<br>M: 53.8 (340)<br>F: 46.2 (292)	1846
Health examination	46.8 (468)<br>M: 35.9 (168)<br>F: 64.1 (300)	37.8 (378)<br>M: 41.0 (155)<br>F: 59.0 (223)	52.0 (520)<br>M: 54.0 (281)<br>F: 46.0 (239)	1366
Included in the present study[2]	46.7 (467)<br>M: 35.8 (167)<br>F: 64.2 (300)	37.8 (378)<br>M: 41.0 (155)<br>F: 59.0 (223)	51.2 (512)<br>M: 42.6 (275)<br>F: 57.4 (237)	1357

[1] Those who participated in the long or short interview and/or the health examination

[2] Those with data for the outcome variable and for at least one of the explanatory variables

M = men, F = women
